# Experiences of reintegration, relapse, and readmission: a qualitative study among patients with alcohol use disorder in Uganda

**DOI:** 10.3389/fpubh.2025.1612634

**Published:** 2025-07-30

**Authors:** Hillary Mugabo Mukula, Morris Ndeezi, Harriet Opondo, Samuel Ouma, Khamisi Musanje

**Affiliations:** ^1^Department of Mental Health and Community Psychology, College of Humanities and Social Sciences, Makerere University, Kampala, Uganda; ^2^Department of Counseling and Higher Education, Ohio University, Athens, OH, United States; ^3^Department of Educational, Social and Organizational Psychology, Makerere University, Kampala, Uganda

**Keywords:** alcohol use disorder, readmission, treatment, relapse, community, reintegration

## Abstract

**Introduction:**

Alcohol Use Disorder (AUD) is a significant public health concern in Uganda, with many patients experiencing relapses. Despite the implementation of various interventions and support services aimed at managing AUD, a substantial number of patients continue to experience relapses, resulting in frequent readmissions. These relapses and readmissions add additional strain to the already burdened healthcare system. Gaining insight into patients' experiences of relapse can help enhance support services and decrease the likelihood of readmissions.

**Methods:**

A descriptive qualitative study examined the relapse experiences of 12 stable AUD patients at Uganda's National Referral Mental Hospital. In-depth interviews were conducted using a semi-structured interview guide, and the data were analyzed thematically through an inductive approach.

**Results:**

Three main themes emerged: causes of relapse, experiences of readmission, and challenges to reintegration. Triggers for relapse included frustration, loneliness, sleep disturbances, negative life events, and peer pressure. Key experiences related to readmission encompassed personal reflection, mastery of treatment, and involuntary admissions. Challenges to reintegration focused on stigma, social disconnection, and feelings of being an outsider.

**Discussion:**

The findings offer valuable insights for enhancing reintegration support services, which may help reduce relapse rates and improve outcomes for AUD patients in Uganda.

## 1 Introduction

Excessive alcohol consumption remains a significant global public health concern, with profound effects on individuals, families, and communities. It contributes to both physical and mental health disorders (1, 2). According to the World Health Organization (WHO) Global Status Report 2018, alcohol use contributes to approximately three million deaths annually (3). WHO statistics further indicate that 43% of the global population aged 15 year and older currently consume alcohol (3, 4). Alcohol Use Disorder (AUD), a substance use disorder marked by excessive drinking despite social, health, psychological, or legal consequences, affects ~1.4% of the global population, or 107 million individuals (5). The global prevalence of AUD at the national level varies between 0.5 and 5% of the general population, with Russia reporting the highest prevalence at 4.7% (6).

In Africa, the prevalence of AUD is estimated at 22%, with Uganda ranking third at 17% (7). Recent global events, such as the COVID-19 pandemic and economic recessions, are believed to have exacerbated the prevalence of AUD (8, 9). The WHO Global Status Report 2023 indicates that Uganda's annual per capita alcohol consumption is 12.21 liters, with a significant gender disparity: men consume an average of 19.93 liters annually, while women consume 4.88 liters (10, 11). Despite the implementation of alcohol regulation laws in Uganda, consumption levels have remained high. Studies show that alcohol initiation occurs as early as 12 years of age among boys, contributing to an increased risk of developing AUD later in life (12).

AUD poses significant socio-economic and health burdens in Uganda. It is associated with reduced productivity, increased household expenditure on alcohol, and elevated risks of intimate partner violence, risky sexual behavior, and road traffic accidents (12–14). A high level of social acceptance of alcohol use in Uganda has further hindered the recognition of alcohol-related health problems, resulting in poor health-seeking behaviors and increased stigmatization of individuals seeking treatment (12, 15). Moreover, AUD treatment in Uganda is primarily focused on acute stabilization and detoxification, with limited access to extensive follow-up care and long-term support services (16). Furthermore, according to the Ministry of Health [Uganda] (17), the pharmacological management of AUD is limited to inpatients in the detoxification stage only, with the Ministry recommending the use of benzodiazepines such as diazepam and phenobarbital, a long-acting barbiturate, as the first-line drugs.

The absence of comprehensive aftercare and follow-up programs significantly increases individuals' vulnerability to relapse, perpetuating a continuous cycle of AUD treatment. Relapse rates remain notably high, pointing out a critical gap in the treatment process and aftercare services (18). Despite the development of various programs aimed at addressing relapses, their persistence suggests that key needs remain unmet (14). Similar studies have highlighted the difficulties patients face when transitioning from inpatient to outpatient care, including limited access to services, difficulty sustaining motivation for sobriety, and challenges with reintegration into family and community life (19, 20). Understanding the lived experiences of patients who experience relapses may provide exceptional insights for tailoring treatment and support services to better meet their specific needs. This study aims to explore the lived experiences of AUD patients in Uganda who have undergone treatment and relapsed.

## 2 Methods

### 2.1 Context

The study was conducted at Butabika National Referral Mental Hospital, Uganda's primary psychiatric facility. The hospital provides outpatient and inpatient psychiatric care, along with specialized services for substance use disorders at the Alcohol and Drug Unit (ADU). This study took place in the ADU, which offers comprehensive treatment for individuals with substance use challenges. The ADU has a private wing and a public wing, both of which admit over 150 patients.

### 2.2 Sampling strategy

The participants were recruited through referrals from the clinical psychologists at the ADU. The potential patients were later contacted, and they voluntarily chose to take part in the study. Participants had to meet three eligibility criteria: a diagnosis of alcohol use disorder (AUD), admission to the ADU, and medical stability to sign consent documents. The research accepted participants who were at least 18 years old and had the ability to communicate in either English or Luganda. Eligible participants were invited for in-depth interviews in therapy rooms at the ADU. Administrative approval was obtained from the ADU administration for the initiation of research prior to commencing recruitment procedures. The study determined the total number of participants using existing research and the concept of data saturation (21). Twelve eligible patients signed consent before joining the research after meeting the study requirements.

### 2.3 Ethical issues pertaining to human subjects

The study was reviewed and approved by the Makerere University School of Health Sciences Research and Ethics Committee (MAKSHSREC-2022-371). For the study to take place at Butabika Hospital, it had to be approved by the Butabika Hospital Research and Ethics Committee (REC-106). A hospital-appointed third party witnessed the signing of the consent forms. Participants provided informed consent and were compensated with UGX10000 for their participation. Data and interviews were de-identified by the first author (HMM). Demographic survey data was not connected to participant interviews or medical records.

### 2.4 Data collection and processing

Interviews were conducted privately in a single session at the Alcohol and Drug Unit (ADU) clinic by the first author (HMM), an experienced qualitative researcher. A semi-structured interview guide was used to explore key themes, including participants' experiences with hospital readmission, factors contributing to relapse, and community reception after discharge. The guide also included open-ended prompts such as: “*Tell me about your experiences with readmission to the hospital,” “What factors contributed to your relapse?”* and “*How did the community receive you after discharge?”* Interviews lasted between 45 and 60 min and were audio-recorded. The recordings were transcribed verbatim by research assistants, with all identifying information removed to maintain confidentiality. Prior to the interview, participants completed a demographic survey that was anonymized and confidential.

### 2.5 Data management and analysis

The study explored participant experiences related to hospital readmission, relapse incidents, and their reintegration into their communities. The research analysis employed thematic methods that integrated both deductive and inductive approaches. The first step involved developing deductive top-level codes derived from the interview guide and categorizing them into three major themes: (1) hospital readmission experiences, (2) factors leading to relapse, and (3) community acceptance after discharge. A trained research assistant conducted a complete, verbatim transcription of all audio documents, and then a native Luganda speaker translated the Luganda transcripts into English. Research participants who provided their contact information received their transcripts for member checking, ensuring the accuracy of the findings. Research data were imported into NVivo 12 software for coding and analysis. The first author, HMM, led the analysis, while the second author, KM, assisted in developing the coding structure. The researchers independently reviewed three transcripts as they generated initial codes using open coding techniques. The approved codebook for the final application was used to analyze all remaining transcripts. Regular team meetings helped enhance the themes and identify newly discovered patterns. The team included direct participant speech verbatim in their analysis while preserving the original language, despite any grammatical errors. The research complies with the Consolidated Criteria for Reporting Qualitative research (COREQ) in presenting its findings.

## 3 Results

### 3.1 Participant demographics

Participants age ranged from 28 to 40 years, with a median of 35.5 years (SD = 4.21). Eleven of the participants identified as males. The average age of onset of alcohol consumption was 16 years. On average, participants reported experiencing three relapses leading to readmission, as shown in Table 1 above.

**Table 1:** Socio-demographic characteristics of participants.

**Demographic characteristics**	***N***	**%**
**Sex**	
Female	1	8.3
Male	11	91.6
**Age**	
25–29	2	17
30–34	3	25
35–39	6	50
40+	1	8
**Number of readmissions**	
0–2	4	34
3–5	7	58
6+	1	8

The study findings are reported following the Consolidated Criteria for Reporting Qualitative Research (COREQ) under three main themes: experiences of reintegration, experiences of readmission and causes of relapse, as shown in Table 2 above.

**Table 2:** Themes and codes identified from the analysis.

**Themes**	**Codes**
Experiences reintegration	Environment
	Stigma
	Lost connection
	Misfit
	Suspicions
Experiences of relapse	Anger
	Frustration
	Sad experiences
	Loneliness
	Boredom
	Lack of sleep
	Peer pressure
	Urge to drink
	Guilt
Experiences of readmission	Personal reflection
	Mastery of treatment
	Nature of treatment
	Involuntary readmissions

### 3.2 Experiences of reintegration

This theme presents experiences of life after discharge from the rehabilitation center. The experiences shared were mainly related to reception upon return to their community, the environment, self-esteem, and challenges of reintegration.

Overall, participants reported experiencing stigma when they returned from rehab. The stigma was instigated by both family members and members of the community in which they lived, and it came in the form of words, nicknames, and jokes used on them. The stigma was further reflected in suspicion whenever they did anything around the home, as exemplified below.

“I don't know whether it's a joke, but some of my family started calling me a mad person and even nicknamed me Boots. Boots, meaning [name of rehabilitation center], I have cousin sisters; I don't think I will ever talk to them again anyway.” (P5 28-year-old male)

“If I want to do something like work, or maybe I want to wash a car or clean up the compound, they will be like, now it is madness that is pushing him to work.” (P11 30-year-old)

“When you get lost, they know you are in rehab, so when you return, they will say ‘you have been with your fellow mad people.' This hits hard.” (P8 28-year-old male)

Furthermore, participants reported losing important connections with friends and family during their stay in rehab. This made reintegration difficult for them, and in the process, they became lonely, as evidenced in the quotes below.

“I lost sensible friends everyone is like you lost your chances eh you get? Sometimes even your parents they no longer trust you so much.” (P6, 34-year-old male)

“I became such a mess that my usual friends, people who I went to school with, no longer needed me. If I go to a party where they are, they throw me out. (P8, 28-year-old male)

“I just wanted somewhere to rest, of course, and some love, but you see, sometimes when you are an addict, no one cares, sometimes you crave for love, you crave for at least a belonging, but everyone has abandoned you. (P6,34-year-old male)

For others, reintegration back into society was difficult because being admitted to rehabilitation automatically made them a misfit. Participants reported how their reputation was soiled because they wore the uniform at the hospital, and for others, they had to prove themselves among their peers, as evidenced below.

“The reputation you also carry when you have worn this uniform also follows you to the outside; you can't fit back.” (P9, 39-year-old male)

“I returned a changed person and committed not to drink again, but my friends would say, how would you fit within the group when taking sodas? At least take [name of alcohol brand], which is lighter, and be a social guy.” (P6 34-year-old male)

“At the point of getting back to the outside, I tried to stay home, keep it on a low. Now, reaching out to friends I was studying with, they were already in their second year, so I was not feeling right because I wasn't fitting in with them.” (P8 28-year-old male)

Many participants' reintegration was characterized by suspicion from significant others. They were checked whenever they left the house, had their rooms checked and for some, they were constantly watched, as exemplified below.

“So, even when I'm moving out, they have to check whether I took anything; even when I come back, they have to see whether I have brought something. I've never had a habit of picking something from home, taking it, or stealing it, but at some point, they said, “I hope you're not picking things from home to go and sell.” (P 5, 28-year-old male)

“Whenever I could do something, they are looking at me, watching curiously; they are like that one anytime he's wires might go off, and this and this and this happens. if I sleep a lot, they are like, huh, maybe he's sick.” (P11, 30-year-old male)

“You see, they start knocking at your door to see if you are not doing anything wrong, so those suspicions, those things for us here, they make us get pissed off. (P1 34-year-old female)

### 3.3 Causes of relapse

When reflecting on the factors that led to their relapse, participants identified anger as one of the factors. Anger was categorized into two: anger toward self, especially after suffering a slip or lapse, and anger toward others that might have led the participants to think of alcohol as a solution, as typified in the quotes below.

“When I took alcohol, I became so angry at myself, that anger in me was pushing me just to go and drink.” (P8, 28-year-old male)

“I was angry with my girlfriend, and the only solution I know was to drink. I know it is not a solution, but, on my side, I said it is the solution.” (P9, 39-years-old Male)

Frustration was also identified to have caused relapse among the participants. Frustration resulted from unfulfilling life and work or mistrust from significant others, who initially took them to the hospital, as exemplified in the quotes below.

“I realized I was overqualified for the job I was doing. I got frustrated and began taking alcohol. I said I should not be doing this job.” (P6, 34-years-old Male)

“I worked so hard, but the reward to what I have studied vis-à-vis what I am doing they did not seem to balance, and the only thing I can tell you that bridging the gap are these substances.” (P7, 39-year-old Male)

“You know how you say, okay now why am I wasting my time? I am trying to sort myself out, but these people do not believe they don't have any trust, so I am wasting my time.” (P1, 34-year-old Female)

All participants reported that they experienced a sad event during or after their discharge that triggered their relapse. These sad events were characterized mainly by loss through separation or death of significant others. Those who experienced the sad event during rehabilitation relapsed immediately after discharge, while those who experienced the event after discharge, relapsed shortly after, as indicated in the excerpts below.

“I lost my mom when I was finishing rehab at [name of treatment facility], and this triggered drinking again.” (P2, 39-year-old male)

“I lost a girlfriend and a brother in the same year and started drinking again.” (P7, 39-year-old, Male)

“I had a girlfriend at my place. When I tested corona positive, I first left her. I went to my sister's place, and when I came back, I told her that I was okay. She said we could not stay together because I was positive. I was not taking alcohol for 9 months, and I started again.” (P6, 34-year-old Male)

Some participants identified loneliness as being a critical trigger to their relapse. Loneliness was most common among participants who had been discharged slightly before and during the COVID-19 lockdown period. Social isolation brought about by the lockdown created the atmosphere for relapse, with many identifying the lack of companionship as being the biggest challenge, as hereto stated.

“I was used to staying with people; now I became lonely, you know, corona period? By six they say you be in the house, and now you are in the house alone and then start drinking.” (P6, 34-year-old Male)

“So, I think at the end of the day I was looking for consolement, someone or something that can console me. And alcohol came in.” (P6, 34-year-old Male)

Relatedly, participants reported boredom as a cause for their relapse. Participants described it as lacking in what to do, especially immediately after discharge or doing the same things after discharge. For many, being bored led them to seek out different ways in which they could keep themselves engaged, and this led to alcohol use in the process, as exemplified in the quotes below.

“So sometimes, the only thing to do is to go to town, sit at a shop, or sit at a bar and start chatting with people, reading newspapers, or looking for jobs in the paper. Because at home, you have workers and there is nothing to do there, sometimes, out of boredom, you find yourself again drinking beer. (P6, 34-year-old Male)

“so, the first start of school, the things they were teaching, I already knew many since I was repeating. So, I was not attending, I got bored, and that is when the devil came in again.” (P8, 28-year-old male)

Peer pressure was also identified as a cause of relapse. Participants described the different ways through which peers in their social circles influenced their decision to resume drinking even after they received the necessary treatment at the alcohol and drug unit. For many, the hospital environment being a restricted area enabled the maintenance of sobriety; however, once discharged, they went back to the same environment, meeting the same friends who ended up encouraging them to drink, as evidenced in the quotes below.

“The war is outside when you are discharged. That's where the war is because we tend to get back to the environment that you were once in, and then you also get to meet still the same people that you were with. Those are the same people who come to visit you, after you are discharged they come and even offer you free drinks because like as if they are celebrating your return.” (P10, 35-year-old Male)

“My friends would say, how would you fit within the group when taking sodas? At least take Tusker Lite, which is lighter, and be a social guy.” (P6, 34-year-old)

“My friends in Kabalagala told me that you can start with Tusker malt and that one might not make you drunk. That is how I started again.” (P.6, 34-year-old)

Some participants identified the lack of sleep as the primary cause of their relapse. They narrated experiencing sleep disturbances as part of the withdrawal symptoms before they were admitted. However, during the admission period, most were given sleeping pills to help them sleep. These were gradually withdrawn to prevent the participants from getting addicted to them. Unfortunately for most, once discharged, they continued to experience these sleep disturbances, and they turned to alcohol as this was the only solution they knew would help them sleep, as indicated in the excerpts below.

“……remember you don't want to drink, but you don't sleep, you try to buy some [name of drug], but still you don't want to get addicted to them, so at the end of it all, you start sliding back, you say ah let me just take only one bottle and I go to bed.” (P1, 34-year-old Female)

“I sometimes used to take alcohol because I wanted to get sleep times I could lose sleep, so that was also one of the factors that made me drink again.” (P10, 35-year-old male)

“When I drink, at least I stabilize and am able to sleep, but when I wake up, I started becoming restless, uncomfortable.” (P8, 28-year-old male)

All participants reported feeling the urge to drink after being discharged from the rehabilitation center. They described the urge to drink as akin to thirst and it was beer to quench the thirst. The body was always demanding more alcohol once they started, and, in the process, they eventually lost control, leading to their relapse, as seen in the quotes below.

“After work, I want to go drink a beer or two, which is normal, but as time goes, my body keeps demanding more and more; that's how everything got worse.” (P7, 39-year-old male)

“……you just drink to satisfy the body and the brain to turn on. The brain goes down, man. Everything is turned upside down minus the drink.” (P2, 39-year-old male)

“The moment you leave alcohol, you have the urge to take something because you are used to taking drinks because it has happened to me, and I started drinking again.” (P6, 34-year-old male)

Overall, participants reported feelings of guilt as being among the factors that led to their relapse. The guilt created a circle of addiction in which the only way to escape from the feeling of guilt was to drink more alcohol and hide from people who knew them, as typified below.

“And at some point, I started hiding from people who knew me; I would now buy and take in cover; I would hide. Because now, sometimes I even would go and drink from another center where they don't know me. Because I felt I was wrong, I was doing something wrong” (P5, 28-year-old male)

“The first one is guilt and shame, and usually you first run, you first try to run away from where you think nobody who knows me is going to be watching.” (P7 39-year-old male)

“Yeah, I would feel guilty, to the extent that I started drinking too much so that I can just sleep off and stop thinking of why I have done this again; something is pushing me like you're doing something wrong.” (P5 28-year-old male)

### 3.4 Experiences of readmission

Under this theme, we report the participants' experiences during readmission, considering the process of readmission, experience with treatment, and period at the hospital. Participants expressed confidence in the rehabilitation center and looked at their stay at the hospital as a moment of reflection and a period to put their lives in order. However, for the majority, readmission was characterized by involuntary admission, which led to resistance and passive participation in the activities at the center, as typified below.

Regarding the process of re-admission, most participants reported that they were forcefully brought back to the hospital against their will, with some being tricked by their relatives. All participants indicated that they hated the involuntary nature of their readmission since they found it humiliating, as indicated in the quotes below.

“They arrested me again and brought me back to [name of center] for the second rehab, then I did three months. Since they had brought me to the rehab when I wasn't using alcohol, that's when I made up my mind to start using alcohol again because, in any case, they can bring me when I have not used, then I started using again, but, but relatively in small amounts.” (P3, 37-year-old male)

“My brother told me there is a ka certain deal I want to crack here in [Name of place]. Can you escort me? I was like, he locked the door. And the next thing we were in [Name of ward], and we found my mother and sister, who had already spoken to the nurse in charge.” (P9, 39-year-old male)

“The first time they brought me here, they just got me from my brother's bar and humiliated me, tied ropes around me and behind me, and they put me in the vehicle behind in the boot, and they brought me here.” (P3, 37-year-old male)

With continued readmission, participants gained mastery of the treatment process. They knew what was going to happen to them at every stage, and some indicated that such processes could even be done from home without necessarily bringing them back to the hospital, as seen in the quotes below.

“Even recently, when I was being brought, I told my brother, why are you taking me back to [name of hospital]? Because I was there… they are just going to give me medicine, which I am even taking from home.” (P12, 40-year-old male).

“… everything was normal. I knew what was going to happen first: detox and then enter inside and chill. (P2, 39-year-old male)

“I said this is the same thing (laughs). I know all the security here. I said do you know what I know the place even if you don't touch me. I know the procedures which we are going to do.” (P9, 39-year-old male)

As time went on, participants began to accept their stay in the rehabilitation center; they opened up to treatment. For many, their stay in the center presented the best time for them to reflect and put their lives in order. It also enabled them to make plans about what to do once discharged, as evidenced below.

“When you come here, you get these sessions, you get these people's stories, you get the psychologist view, and so you start contemplating about your life.” (P1 34-year-old female)

“I started to realize how much I'm hurting my family. It was a big step for me to take. I used sometimes to sit down and reflect upon what someone has said during a session, and I would sometimes think that maybe alcohol is not my thing.” (P8 28-year-old male)

“I detached from the world I was living in; it put me in some controlled environment for me to see how I am without the rest of whatever it is inside here; it woke me up to the fact that I can be someone or something very big if I did away with the substances.” (P7 39-year-old male)

Finally, participants also discussed the nature of the treatment they received. They reported having gone through a process of detoxification, which involved the use of medication, and stabilization, which involved therapeutic sessions characterized by group and individual sessions with psychologists and counselors. With continued readmission, participants came to validate the treatment and identified what worked well for them and what did not during the treatment period, as elucidated below.

“Talking is the best treatment because anyone can get the medicine out there; even someone can come and get prescribed and go home and take that medicine. If that medicine was helping us leave alcohol, then we would come and buy it from pharmacies and take it home and stop. But I think the talking, I think this therapy of talking is more important.” (P6, 34-year-old Male)

“Talking to a counselor was helpful because when I would share and say what I've been thinking to someone like a psychologist, not a fellow patient, they would not say what I've thought of is wrong.” (P8, 28-year-old male)

“Treatment is always like it can take you like three weeks, then afterward they get you off treatment, then you remain with therapy; therapy is not bad; they take you through different challenges that you are faced through.” (P10 35-year-old male)

## 4 Discussion

Our study became one of the earliest efforts to highlight the perspectives of patients diagnosed with alcohol use disorder (AUD) in Uganda. Several novel findings emerged; our results indicated that patients diagnosed with AUD perceive relapse as an ongoing habit influenced by emotions and their environment. During treatment and readmission, participants shared their struggles, including being admitted against their will and resisting treatment before eventually accepting rehabilitation. Additionally, many participants expressed finding it difficult to return to their previous lives, and community discrimination against them as former addicts made it challenging to maintain recovery sobriety. Our research extends knowledge about Alcohol Use Disorder treatment experiences in low-resource areas by presenting insights from individuals as they transition through treatment and reintegration in Uganda.

It is generally known that reintegration is a vulnerable period for individuals recovering from AUD, with a high risk of relapse due to emotional and social stressors (22). We found that the process of reintegration back into the community following discharge from rehabilitation was crucial to participants' ability to maintain sobriety. Our research highlights how stigma hinders participants‘ return to everyday life after rehabilitation, making it difficult to achieve and maintain sobriety. Participants reported feeling excluded and labeled by their community, which hindered their efforts to reintegrate and worsened their self-image. Additionally, study participants agreed that they faced triggers, including social isolation and peer influence, a finding similar to previous studies about alcohol use disorder recovery (23, 24). Furthermore, our research findings identify strategies that can guide improvements in the real-world substance abuse treatment framework. Research indicates that when individuals receive structured vocational training and support from peers, they experience greater community integration after completing rehabilitation (25, 26). Without a proper reintegration plan, relapse is likely to happen (27).

Studies on alcohol dependence and addictive behaviors reveal notoriously high relapse rates, indicating that maintaining behavioral change over time and across situations is a problematic issue (28). Relapse creates intense discomfort for both individuals with AUD and everyone involved in their care. The impact of relapse feels intense because patients and everyone involved experience crushing disappointment in their combined efforts to maintain sobriety (29, 30). Study findings from Butabika National Referral Mental Hospital show similar reasons for AUD patients' relapse (18). We found that frustration was a significant factor contributing to relapse among participants. Many described feelings of dissatisfaction with their personal and professional lives, which created a sense of hopelessness and ultimately drove them back to alcohol use. Participants reported frustration with their inability to secure meaningful employment or achieve their life goals, leading to feelings of stagnation and disappointment. This frustration was compounded by a perceived lack of trust and support from their families and social circles, further reinforcing feelings of isolation and failure. Similar findings have been reported in previous studies, which highlight how persistent frustration can erode an individual's motivation to stay sober and increase their vulnerability to relapse (31, 32).

Participants revealed that their relapse following rehabilitation programs occurred from habit-related behavioral problems coupled with emotional distress and personal relationship challenges. They shared that they feared their old drinking zones after treatment and felt tempted to follow normal social drinking habits. Research by Kabwama et al. found that the chances of relapse increase due to both external factors and personal barriers to remaining sober (14). Participants also indicated a need for improved support programs, noting that treatment facilities did not adequately teach effective coping methods or provide proper follow-up evaluations. Future research must examine whether incorporating peer support alongside planned discharge strategies in rehabilitation affects relapse rates and assists patients in maintaining sobriety after treatment. Existing research indicates that the period following discharge is crucial for recovery from Alcohol Use Disorder (AUD) (33–35). This study's findings reveal that patients are frequently re-admitted due to limited follow-up care after discharge, yet most studies focus on relapse rather than on readmission.

Only a few studies have focused specifically on readmission. However, a recently published study found that 50.8 percent of 132 older male alcoholics were readmitted to detoxification within 1 year of discharge (36). We have not found any studies focusing on patients' experiences of readmission in Uganda. Some key findings from our study highlight the methods used for readmission, which include deception, forced arrest, and coercion. Since the only government-funded alcohol and drug treatment unit is located within a mental hospital, participants expressed reluctance to voluntarily enter the unit due to the stigma associated with the hospital. The findings indicate that the primary concern among participants regarding their hospitalization was the coercive nature of their re-admission. The lack of involvement in decision-making during the process led to resistance toward participating in treatment, which manifested as reluctance to take medication, tendencies toward self-isolation, and attempts to escape. This finding confirms the concerns raised by previous scholars about the long-term effects of involuntary admission on patients, including its impact on their psychological wellbeing (37, 38). Furthermore, previous scholars have found that involuntary admission is a predictor of relapse among patients with alcohol use disorder (39, 40). Care for involuntarily admitted patients is complex due to the resistance of the patients. However, findings from this study indicate that the effects of involuntary admission can be mitigated with the provision of a peer support system and therapeutic sessions that provide hope to the patients. Similar to previous studies, peer support systems and hope are effective when treating patients with alcohol use disorder (41, 42). Our study also found that the treatment of alcohol use disorder is characterized by stabilization and detoxification, which involves the use of medication. Psychotherapy sessions focus solely on assessing readiness for discharge, with minimal comprehensive follow-up care and support (16). Our findings indicate that participants believe psychotherapy sessions are essential because they provide hope. Existing literature examining hope among individuals undergoing treatment for substance use disorders has found that patients with high levels of hope are better prepared to face challenges during and after treatment (43). However, hope does not operate independently; a supportive environment is another critical aspect of recovery. While in the hospital, our study found that interacting and sharing experiences with peers was beneficial, with some participants only appreciating the treatment after moving from the private ward to the general ward with more people. This further highlights the significance of social support in the recovery process. As reported in previous studies, an alcoholic who is not marginalized or stigmatized in the ward integrates well with fellow members and is likely to have better outcomes and abstain (44).

## 5 Conclusion

Overall, our findings expand on and reinforce the existing literature regarding the lack of transitional support and limited access to aftercare services for individuals recovering from AUD after discharge from rehabilitation. They also underscore the necessity for comprehensive relapse prevention strategies by highlighting the suggestions made by individuals with lived experience of AUD who encountered significant challenges in maintaining sobriety post-rehabilitation. The post-discharge period presents a heightened risk of relapse, making improved access to aftercare support during this time crucial for reducing the recurrence of alcohol use and related health complications. A lack of consistent follow-up care and engagement with treatment services may contribute to relapse, undermining recovery efforts and increasing the likelihood of repeated readmissions. Researchers advocate for developing multi-level interventions that span individual, programmatic, and societal levels to bridge the gap during the reintegration process and enhance recovery outcomes. Transitional programs, connections to outpatient counseling services, and reintegration interventions like case management have been linked to better treatment retention and lower relapse rates in individuals with AUD.

## 6 Limitations

Despite the numerous positive contributions, this study had some limitations. Participants were sampled from the National Referral Mental Hospital; this selection criterion may have excluded individuals who experienced relapse but did not want to be readmitted due to the location of rehabilitation center. Furthermore, most participants were awaiting discharge, which may have influenced some of their responses. This social desirability bias might have led some participants to alter their narratives, downplaying challenges or exaggerating successes with treatment. However, this bias may have been mitigated by the researchers focusing on the patients' retrospective experiences of reintegration, readmission, and relapse. Future studies should consider conducting a similar study with participants who are not institutionalized, who have received treatment, have been discharged, and have experienced relapse.

## Data Availability

The raw data supporting the conclusions of this article will be made available by the authors, without undue reservation.
