# Supplementary material for: Experiences of reintegration, relapse, and readmission: a qualitative study among patients with alcohol use disorder in Uganda
Source: Front Public Health. 2025 Jul 30;13:1612634. doi: 10.3389/fpubh.2025.1612634 (PMC12343713; doi:10.3389/fpubh.2025.1612634)
Supplement: Supplementary file 1 [file Table_1.docx]

Interview Guide

Introduction: We are conducting interviews to better understand the experiences of relapse, treatment and reintegration among people diagnosed and treated for alcohol use disorder in Uganda. I will spend 1-hour with you. To help us get started, I have a series of questions that I will be asking you. There is no right or wrong answer, the most helpful information is when you provide your honest opinions and personal experiences. I want to you that I will save the audio recording files of this interview. Please respond to all questions, however, you are free to skip a question in case it makes you uncomfortable. Your name and identity will be concealed and not tied to any of your responses. Feel free to interrupt or ask questions at any point in the interview.

Can we start? <To proceed only when the Respondent answers positively>

1. Tell me about your experiences with alcohol use before seeking treatment?

Probes

i. What made you stop consuming alcohol?

ii. What made you seek treatment for alcohol use?

2. Tell me about the treatment you received the first times you were admitted?

Probes

i. What in your opinion worked well for you?

ii. What in your opinion was not helpful for you?

3. Tell me about your experiences after treatment and back in the community?

Probes

i. How did you feel finally going back home after treatment?

ii. How were you received by your family, friends and the community?

iii. What are some of the good and bad experiences you encountered when you returned to your community

4. Tell me about your experiences after you relapsed?

Probes

i. What do you think led to your relapse?

ii. How did you feel when you started consuming alcohol again?
